# RETRACTION: Aβ‐Induced Repressor Element 1‐Silencing Transcription Factor (REST) Gene Delivery Suppresses Activation of Microglia‐Like BV‐2 Cells

**DOI:** 10.1155/np/9758713

**Published:** 2026-05-12

**Authors:** Neural Plasticity

RETRACTION: T. Yu, H. Quan, Y. Xu, et al., “Aβ‐Induced Repressor Element 1‐Silencing Transcription Factor (REST) Gene Delivery Suppresses Activation of Microglia‐Like BV‐2 Cells,” *Neural Plasticity*, 2020, 8888871, https://doi.org/10.1155/2020/8888871.

The above article, published online on 22 September 2020 in Wiley Online Library (wileyonlinelibrary.com), has been retracted by agreement between the Journal Editor‐in‐Chief, Michel Baudry; and John Wiley & Sons Ltd. The retraction has been agreed due to errors in the figures as follows:

• The graph in Figure 2b is identical to the graph in Figure 2b of [1] by some of the same authors, despite the western blots appearing different
• The REST bands in Figure 3a are vertically and horizontally rotated versions of the 0µM and 1 µM REST bands in Figure 2c of [1]
• The graph in Figure 3b is identical to graph in Figure 4a of [1], despite the western blots appearing different
• The 5µM Aß panels in Figure 3d appear identical to Figure 4e of [1]

Following these concerns being raised, the authors were unable to provide the raw data for the study and therefore requested the retraction of the article. The article is therefore retracted with the agreement of the Editorial Board and the authors.
